# Preoperative predictors of adverse pathology and recurrence‐free survival for patients with renal masses

**DOI:** 10.1002/bco2.70175

**Published:** 2026-02-27

**Authors:** Akira Kazama, Carlos Munoz‐Lopez, Worapat Attawettayanon, Eran Maina, Nityam Rathi, Kieran Lewis, Anne Wong, Angelica Bartholomew, Rebecca A. Campbell, Jihad Kaouk, Samuel Haywood, Nima Almassi, Christopher J. Weight, Nick Heller, Shetal Shah, Erick M. Remer, Ryan Ward, Amy S. Nowacki, Steven C. Campbell

**Affiliations:** ^1^ Glickman Urological and Kidney Institute Cleveland Clinic Cleveland Ohio USA; ^2^ Department of Urology, Molecular Oncology Niigata University Graduate School of Medical and Dental Sciences Niigata Japan; ^3^ Division of Urology, Department of Surgery, Faculty of Medicine, Songklanagarind Hospital Prince of Songkla University Songkhla Thailand; ^4^ Imaging Institute Cleveland Clinic Cleveland Ohio USA; ^5^ Department of Quantitative Health Sciences Cleveland Clinic Cleveland Ohio USA

**Keywords:** adverse pathology, nomogram, parenchymal volume replacement, partial nephrectomy, radical nephrectomy, renal cell carcinoma

## Abstract

**Objectives:**

Our objective was to develop algorithms to predict adverse pathology (AP) and recurrence‐free survival (RFS) for patients with renal tumours primarily based on multifaceted analysis of preoperative CT imaging.

**Patients/Methods:**

Seven hundred forty‐eight patients with non‐metastatic renal tumours managed with definitive surgery at Cleveland Clinic (2011–2014) were retrospectively evaluated (median follow‐up 9.1 years). All patients underwent contrast‐enhanced CT and parenchymal volume analysis using semi‐automated software. A variety of conventional radiological features were evaluated in addition to parenchymal volume replacement (PVR) due to invasive tumour growth, using the contralateral kidney as a control. Adverse pathology (AP) was defined as stage ≥pT3a, grade 3/4 or sarcomatoid/rhabdoid features. Multivariable logistic regression and Cox proportional hazards regression analyses were used to develop predictive models.

**Results:**

Overall, 339/748 patients (45%) had AP, which significantly associated with reduced RFS. On univariable analysis, tumour‐size, degree of vascularity, heterogeneity, irregular contour, sinus margin irregularity, necrosis, non‐cystic tumour and increased PVR significantly associated with AP. On multivariable logistic regression, male sex, R.E.N.A.L. Nearness, heterogeneity, necrosis, sinus margin irregularity and PVR ≥ 25% independently associated with AP. Multivariable analysis indicated that tumour size, heterogeneity, necrosis, PVR ≥ 25% and tumour‐related symptoms significantly associated with reduced RFS. Models for AP and RFS at 3, 5 and 10 years showed area under the curve (AUC) values of 0.81 and 0.84–0.86, respectively.

**Conclusions:**

These findings confirm that radiological features and PVR are associated with AP and reduced RFS after definitive renal cancer surgery. Our predictive models are entirely based on preoperative parameters and may improve patient counselling and occasionally preclude the need for renal mass biopsy.

## 1 INTRODUCTION

The primary treatments for localized renal tumours are surgical excision by radical nephrectomy (RN) or partial nephrectomy (PN), percutaneous thermal ablation (TA) or active surveillance (AS).^1^, ^2^ Decisions regarding the management of this patient population can be complex, with functional and oncologic implications and varying risks of perioperative morbidity.^1^, ^2^, ^3^ Traditionally, such choices have been made through careful counselling and shared decision‐making, with consideration of tumour size and complexity, baseline glomerular filtration rate and assessment of patient age, comorbidities and life expectancy.^1^, ^2^, ^3^, ^4^ Renal mass biopsy (RMB) has typically been used whenever additional preoperative oncologic risk stratification might be of utility, but RMB is an invasive procedure that is only diagnostic in about 85%–90% of patients.^1^, ^2^, ^5^, ^6^ Ideally, overtreatment would be avoided for indolent or benign tumours, and improved knowledge of the aggressive potential of the mass can influence decisions about PN versus RN, wide margin PN versus tumour enucleation and TA versus PN versus AS for patients with tumours on the less aggressive end of the spectrum.^1^, ^2^, ^3^, ^7^, ^8^

Many studies have been reported to predict adverse pathology (AP) using risk assessment models based on patient and tumour characteristics and preoperative radiological features.^9^, ^10^, ^11^, ^12^, ^13^, ^14^, ^15^, ^16^, ^17^, ^18^, ^19^ Contrast‐enhanced CT scans are the most common imaging modality used to evaluate renal tumours, to discriminate between benign and malignant histology and to predict pathologic determinations such as tumour grade or pT3a upstaging.^1^, ^2^, ^3^ Many previously published models combined general patient and tumour‐related features, including age, sex, R.E.N.A.L. nephrometry score and tumour size,^9^, ^10^, ^11^, ^12^, ^13^ which has traditionally proven to be the most impactful predictor. In some of these studies, radiologic features have also been incorporated including degree of enhancement or heterogeneity and assessment of the tumour contour, and some have also incorporated pathologic features,^14^, ^15^, ^19^ thereby limiting their utility for preoperative counselling.

Our objective was to provide a comprehensive evaluation of radiologic features to optimize preoperative risk stratification for patients with renal masses managed with definitive renal cancer surgery. Our main outcome parameters are AP, defined as stage ≥pT3a, tumour grade 3/4 or sarcomatoid/rhabdoid features, and recurrence‐free survival (RFS), which was facilitated by a median overall survival of 9 years in our cohort. We also include assessment of parenchymal volume replacement (PVR) due to invasive tumour growth, which leads to reduced parenchymal volumes in the ipsilateral kidney.^20^ PVR has correlated with oncologic outcomes in other studies.^21^ Our goal is to improve preoperative oncologic risk stratification and to potentially preclude the need for RMB for some patients.

## 2 PATIENTS AND METHODS

### 2.1 Patient population

A comprehensive retrospective review was performed of the Cleveland Clinic kidney cancer database after Institutional Review Board approval (IRB‐20‐836). Of 2336 patients with localized kidney tumours managed with PN or RN in the timeframe of 2011–2014, 748 patients had all necessary studies and were evaluated. Inclusion required age greater than 18 years and current availability of preoperative contrast‐enhanced CT imaging within 3 months of surgery—those with MRI or ultrasound rather than CT were excluded. Patients with a solitary kidney, multiple or bilateral synchronous renal tumours, tumours with a distinct fat component and kidneys with substantial focal scarring or infarct (involving over 10% of either kidney) or global contralateral renal atrophy (renal length less than 8 cm) were also excluded. The latter exclusion criteria, which represented <2% of the total population, were excluded because atrophy or infarct could distort calculations of PVR (see below).

### 2.2 Data collection

Baseline characteristics including demographics, comorbidities and tumour characteristics were collected. Pathological findings including tumour grade, histology and stage were recorded with stage classified according to 2016 American Joint Committee on Cancer/Union for International Cancer Control TNM.^22^ AP was defined as stage ≥pT3a, tumour grade 3/4 or sarcomatoid/rhabdoid features. Contrast‐enhanced CT images were blindly and independently evaluated by two urologists (A.K. and C.M.L., with mentoring and guidance from radiologists, E.M.R. and R.W.) for the following seven traditional features: tumour size, enhancement (hypovascular vs. hypervascular), heterogeneity (homogeneous vs. heterogeneous), tumour contour (smooth vs. lobular/infiltrative), tumour sinus margin (regular vs. irregular), presence of necrosis and cystic tumour (cystic features >25%) (Figure S1). When there was discordance between the two reviewers, the images were reviewed by the group (A.K., C.M.L. and S.C.C./E.M.R./R.W.) to achieve consensus.

### 2.3 Parenchymal volume replacement (PVR)

**FIGURE 1:**
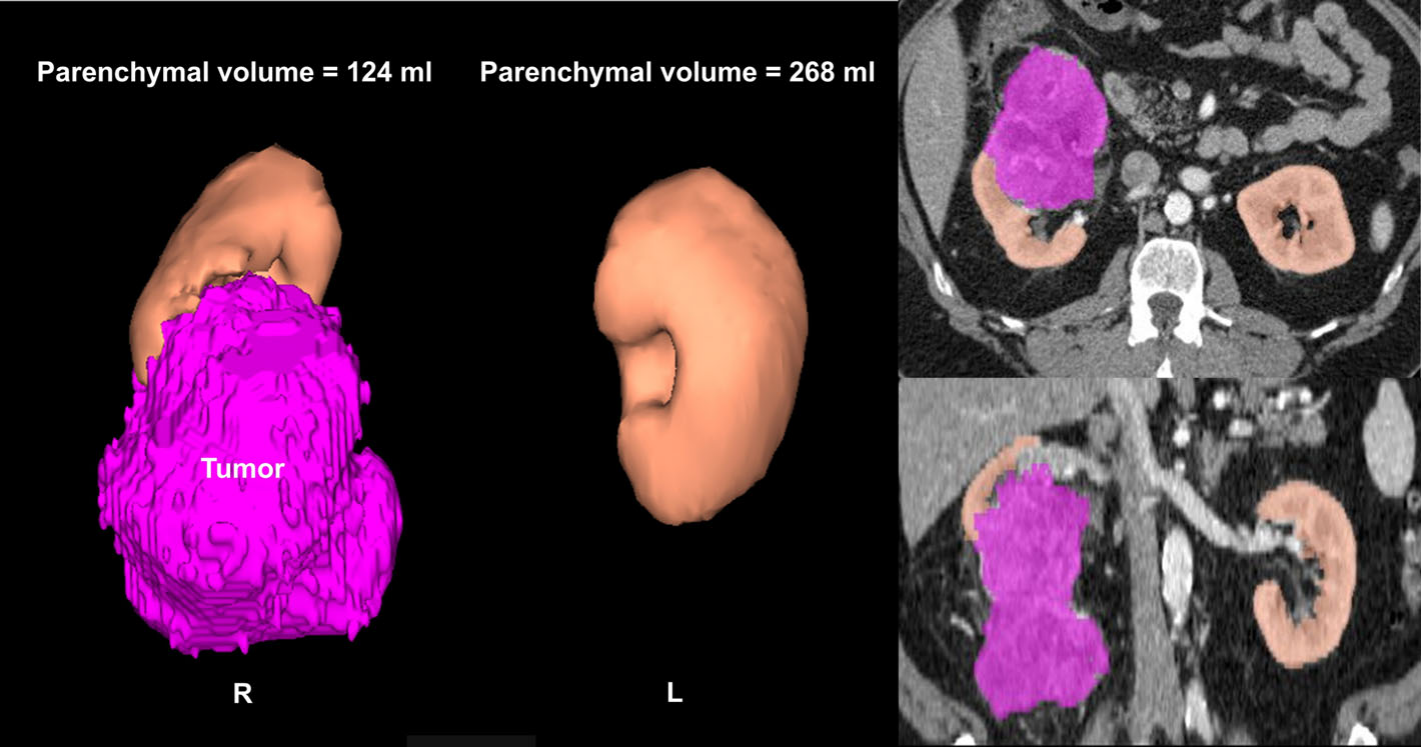
Estimation of parenchymal volume replacement (PVR). Parenchymal volume replacement was estimated using semi‐automated software with the tumour shown in purple and the parenchyma in beige. The images on the left show three‐dimensional views of the kidneys and on the right images from the transverse and coronal sequences with parenchymal volumes overlayed on the standard CT image. Percent PVR is defined as parenchymal volume_contralateral_ − parenchymal volume_ipsilateral_ normalized by parenchymal volume_contralateral._ ^20^, ^21^ In this case, the parenchymal volumes in the left and right kidneys were 268 and 124 mL, respectively. The PVR value was then [(268 − 124)/268] × 100% = 54%.

Parenchymal volume analysis was performed using semi‐automated three‐dimensional volume calculating software (SYNAPSE 3D®, Fujifilm Medical Systems, Figure 1).^23^, ^24^ Presuming that the contralateral kidney serves as a control, percent PVR was defined as

$$\frac{\left({\text{Parenchymal Volume}}_{\text{contralateral}}‐{\text{Parenchymal Volume}}_{\text{ipsilateral}}\right)}{{\text{Parenchymal Volume}}_{\text{contralateral}}}\times 100\%$$

Percent PVR was classified by degree: minimal (<5%), modest (5%–25%) and prominent (≥25%) based on previous studies, which showed that PVR correlated with oncologic outcomes.^20^, ^21^

### 2.4 Statistical analyses

Continuous variables were presented as medians with interquartile range and compared using Mann–Whitney *U*‐test. Categorical variables were presented as frequencies (*N*) with percentages and compared using Fisher's exact test. Cohen's kappa was used to quantify the interrater reliability between initial CT imaging evaluation of two investigators. A Kaplan–Meier curve was constructed to estimate RFS with log‐rank test assessing differences among the cohorts with AP versus those without AP.

Multivariable logistic regression and Cox proportional hazards regression modelled predictors of AP and RFS, respectively. To construct the models for prediction of AP or RFS, candidate variables with *p* < 0.10 in univariable analyses were initially entered into the model. A backward stepwise selection procedure was then applied, sequentially removing variables with the highest *p* values until all remaining predictors met the retention threshold of *p* < 0.05. In addition to statistical significance, clinical relevance was also considered during the selection process. Nomograms and receiver operating characteristic (ROC) analyses were generated based on logistic regression models for predicting AP and Cox proportional hazards models for RFS. Model discrimination was evaluated using Harrell's concordance index (C‐index) with 95% confidence intervals. The R code used to generate the logistic regression and Cox proportional hazards models, along with the corresponding nomograms and time‐dependent ROC analyses based on the Cox model, is provided in Table S3. A calibration curve with bootstrap resampling was used to validate the accuracy of the predictive model.

All tests were two‐tail; *p* < 0.05 was considered statistically significant. All statistical analyses were performed with R statistical software version 4.3.2 (R Foundation Statistical Computing).

## 3 RESULTS

### 3.1 Patient and tumour characteristics

Overall, 748 patients met the inclusion criteria, and baseline characteristics are summarized in Table 1. Median age was 63 years and 484 (65%) were male. Eighty‐five percent of the patients were Caucasian, and the median BMI was 30 kg/m^2^. Median tumour size and R.E.N.A.L. scores were 4.3 cm and 8, respectively. PN was performed in 63% of the patients and RN in 37%. Locally advanced renal cancer (≥pT3) was found in 25% of the patients, benign histology in 12%, tumour grade was 3/4 in 49% of the malignant cases and sarcomatoid/rhabdoid features were found in 3.3%. Overall, AP was found in 339 (45%) patients. Based on the AP diagnostic criteria, 193 cases met only one criterion (≥pT3; *n* = 49, tumour grade 3/4; *n* = 144, sarcomatoid; *n* = 0), 128 met two criteria (≥pT3 and tumour grade 3/4; *n* = 121, ≥pT3 and sarcomatoid; *n* = 0, tumour grade 3/4 and sarcomatoid; *n* = 7) and 18 met all three criteria. The median follow‐up was 9.1 years.

**TABLE 1:** Patient and tumour characteristics.

Parameter	Overall (*N* = 748)^a^
Age (year)	63 [54, 70]
Male	484 (64.7%)
Race	
Caucasian	631 (84.7%)
African‐American	93 (12.5%)
Other	21 (2.8%)
BMI (kg/m^2^)	30 [26, 35]
Hypertension	447 (59.8%)
Diabetes	162 (21.7%)
Cardiovascular disease	180 (24.1%)
Charlson score	4 [3, 6]
Smoking	384 (52.0%)
Tumour size (cm)	4.3 [2.7, 6.4]
R.E.N.A.L. score	8 [6, 10]
R.E.N.A.L. complexity	
Low (R.E.N.A.L. 4–6)	211 (28.2%)
Intermediate (R.E.N.A.L. 7–9)	294 (39.3%)
High (R.E.N.A.L. 10–12)	243 (32.5%)
Nephrectomy	
Partial	471 (63.0%)
Radical	277 (37.0%)
Technique	
Open	277 (37.0%)
Robotic/laparoscopic	471 (63.0%)
EBL (mL)	200 [100, 350]
Complications (grades 3–5)	43 (5.7%)
pT stage	
T1a	303 (40.8%)
T1b	131 (17.6%)
T2a/b	29 (3.9%)
≥T3a	188 (25.3%)
Benign	92 (12.4%)
Histology	
Clear cell	457 (61.1%)
Papillary	114 (15.2%)
Chromophobe	52 (7.0%)
Other RCC	28 (3.7%)
Other cancer	4 (0.5%)
Benign	93 (12.4%)
Pathological tumour grade	
Grade 1/2	307 (51.4%)
Grade 3/4	290 (48.6%)
Sarcomatoid/rhabdoid feature	25 (3.3%)
Adverse pathology^b^	339 (45.3%)
Positive surgical margin	41 (5.5%)

Abbreviations: BMI = body mass index; EBL = estimated blood loss; *N* = number; pT stage = pathological tumour stage; R.E.N.A.L. = [R]adius, tumour size as maximal diameter; [E]xophytic/endophytic properties of tumour; [N]earness of tumour deepest portion to collecting system or sinus; [A]nterior [a]/posterior [p] descriptor; and [L]ocation relative to polar line.

^a^ Summary statistics are reported as either *N* (%) or median [Q_1_ − Q_3_].

^b^ Adverse pathology was defined as stage ≥pT3a, grade 3/4 or sarcomatoid/rhabdoid features.

### 3.2 Predictive model for AP

**FIGURE 2:**
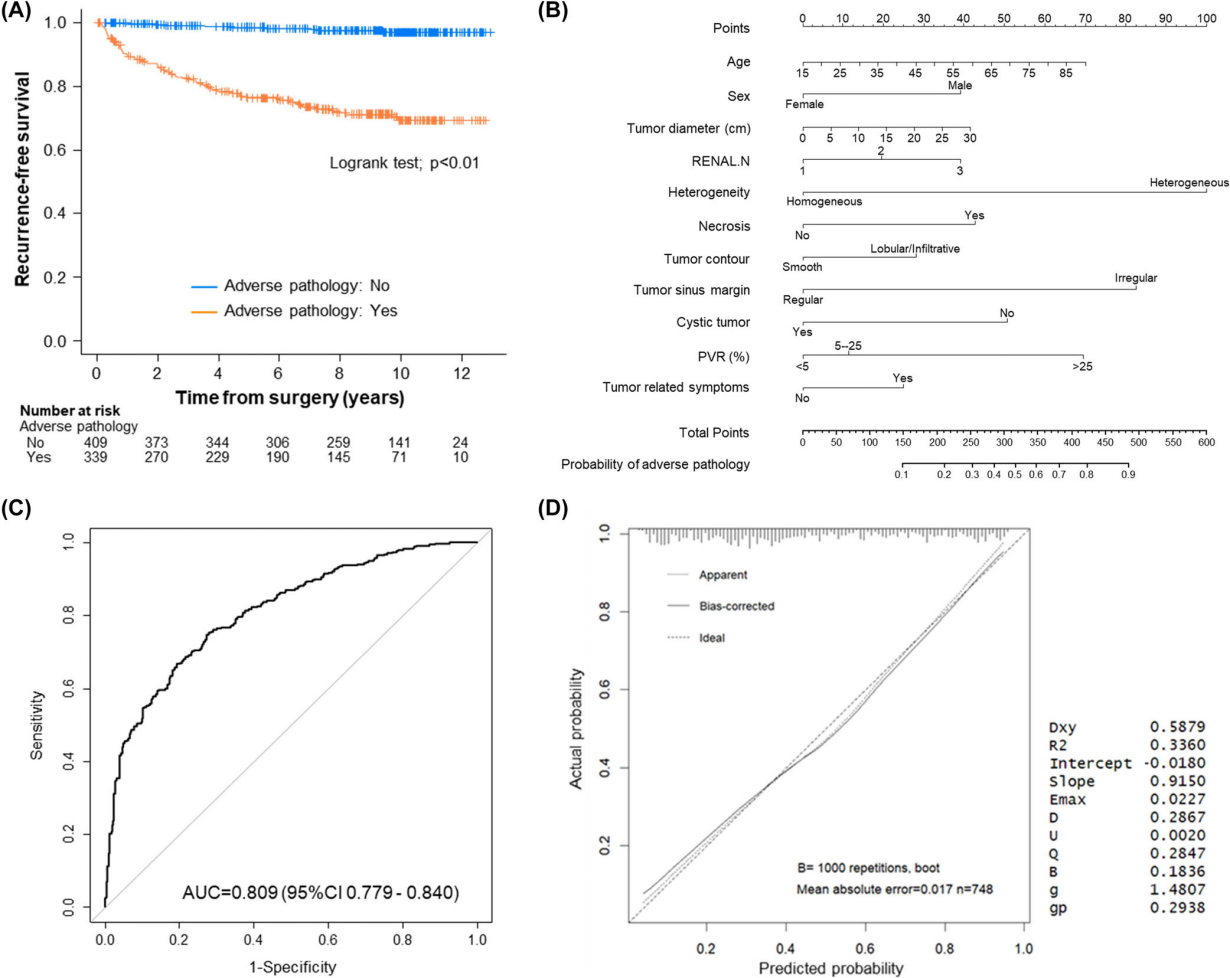
Impact of adverse pathology (AP) and predictive models. (A) Kaplan–Meier curves showing the recurrence‐free survival probability for time after surgery for patients with non‐adverse pathology (*n* = 409; blue) versus patients with adverse pathology (*n* = 339; orange). Comparisons between the adverse pathology and non‐adverse pathology cohorts were evaluated by log‐rank test. (B) Nomogram to predict the probability of adverse pathology derived from a multivariable logistic regression model based primarily on imaging features. (C) ROC curve derived from the nomogram (apparent C‐index = 0.809, bias‐corrected C‐index = 0.795). (D) A calibration curve comparing predicted and actual event probabilities. AUC = area under the curve, PVR = parenchymal volume replacement, R.E.N.A.L. = [R]adius, tumour size as maximal diameter; [E]xophytic/endophytic properties of tumour; [N]earness of tumour deepest portion to collecting system or sinus; [A]nterior [a]/posterior [p] descriptor; and [L]ocation relative to polar line, ROC = receiver operating characteristic.

On univariable analysis, age, tumour size, R.E.N.A.L. score, presence of tumour related symptoms, degree of vascularity, heterogeneity, irregular contour, sinus margin irregularity, necrosis, non‐cystic tumour and increased PVR all significantly associated with AP (all *p* < 0.01, Table 2). Interrater reliability for all radiological features were strong (all κ values > 0.85, Table S1). On multivariable logistic regression analysis, male sex, R.E.N.A.L. Nearness score, heterogeneity, necrosis, sinus margin irregularity and prominent PVR (≥25%) independently associated with AP (Table 3). As shown in Figure 2A, AP was significantly associated with reduced RFS (*p* < 0.01).

**TABLE 2:** Patient/tumour characteristic and preoperative radiological features.

Parameter	Non‐adverse pathology^a^ (*N* = 409)	Adverse pathology^a^ (*n* = 339)	*p* value
Age (year)	60 [51, 69]	65 [56, 71]	<0.01
Male	254 (62.1%)	230 (67.8%)	0.11
Race			0.99
Caucasian	343 (84.3%)	288 (85.2%)	
African‐American	52 (12.8%)	41 (12.1%)	
Other	12 (2.9%)	9 (2.7%)	
BMI (kg/m^2^)	30 [26, 35]	30 [26, 35]	0.95
Tumour size (cm)	3.4 [2.2, 5.0]	5.5 [3.6, 8.2]	<0.01
R.E.N.A.L. score	7 [5, 9]	10 [8, 11]	<0.01
Tumour‐related symptoms^b^	26 (6.4%)	62 (18.3%)	<0.01
Enhancement			<0.01
Hypovascular	140 (34.2%)	65 (19.2%)	
Increased vascularity	269 (65.8%)	274 (80.8%)	
Heterogeneity			<0.01
Homogeneous	116 (28.4%)	18 (5.3%)	
Heterogeneous	293 (71.6%)	321 (94.7%)	
Necrosis	62 (15.2%)	159 (46.9%)	<0.01
Contour			<0.01
Smooth	241 (58.9%)	93 (27.4%)	
Lobular/infiltrative	168 (41.1%)	246 (72.6%)	
Sinus margin			<0.01
Regular	363 (88.8%)	172 (50.7%)	
Irregular	46 (11.2%)	167 (49.3%)	
Cystic			<0.01
Cystic	241 (58.9%)	93 (27.4%)	
Non‐cystic	168 (41.1%)	246 (72.6%)	
Parenchymal volume replacement^c^ continuous (%)	3.4 [−4.5, 11.1]	11.4 [1.0, 25.6]	<0.01
Parenchymal volume replacement^c^ categorical			<0.01
<5%	240 (67.4%)	116 (32.6%)	
5%–25%	143 (51.3%)	136 (48.7%)	
≥25%	26 (23.0%)	87 (77.0%)	

*Note*: Summary statistics are reported as either *N* (%) or median (Q_1_ − Q_3_).

Abbreviations: BMI = body mass index; GFR = glomerular filtration rate; *N* = number, R.E.N.A.L. = [R]adius, tumour size as maximal diameter; [E]xophytic/endophytic properties of tumour; [N]earness of tumour deepest portion to collecting system or sinus; [A]nterior [a]/posterior [p] descriptor; and [L]ocation relative to polar line.

^a^ Adverse pathology was defined as stage ≥pT3a, grade 3/4 or sarcomatoid/rhabdoid features.

^b^ Tumour‐related symptoms include flank/abdominal pain, gross haematuria, abdominal mass, weight loss >10% or loss of appetite, anaemia and severe fatigue.

^c^ Percent parenchymal volume replacement = [(parenchymal volume_contralateral_ − parenchymal volume_ipsilateral_) normalized by parenchymal volume_contralateral_] × 100%.

**TABLE 3:** Multivariable analysis of predictors of adverse pathology and recurrence‐free survival.

Logistic regression analysis for adverse pathology^a^		
Predictor	Odds ratio (95% CI)	*p* value
Age (year)	1.01 (0.99, 1.03)	0.09
Sex [male]	1.67 (1.15, 2.42)	**<0.01**
Tumour diameter (cm)	1.02 (0.94, 1.11)	0.68
R.E.N.A.L. N score	1.29 (1.02, 1.63)	**0.03**
Tumour‐related symptoms^b^ [yes]	1.39 (0.76, 2.52)	0.28
Heterogeneity [heterogeneous]	3.70 (2.07, 6.64)	**<0.01**
Necrosis [yes]	1.75 (1.12, 2.72)	**0.01**
Tumour contour [lobular/infiltrative]	1.44 (0.99, 2.13)	0.06
Tumour sinus margin [irregular]	2.95 (1.87, 4.65)	**<0.01**
Cystic tumour [yes]	0.51 (0.26, 1.03)	0.06
PVR^c^		
[<5%]	Reference	
[5%–25%]	1.16 (0.80, 1.70)	0.44
[≥25%]	2.48 (1.30, 4.73)	**<0.01**

Cox‐regression analysis for recurrence‐free survival (RFS)		
Predictor	Hazard ratio (95% CI)	*p* value
Age (year)	1.00 (0.98, 1.01)	0.61
Sex [male]	1.42 (0.92, 2.21)	0.12
Tumour size (cm)	1.06 (1.00, 1.12)	**0.047**
R.E.N.A.L. N score	1.14 (0.76, 1.69)	0.53
Tumour‐related symptoms^b^ [yes]	2.25 (1.44, 3.53)	**<0.01**
Heterogeneity [heterogeneous]	4.29 (1.01, 18.2)	**0.049**
Necrosis [yes]	2.41 (1.46, 3.98)	**<0.01**
Tumour contour [lobular/infiltrative]	1.15 (0.62, 2.13)	0.66
Tumour sinus margin [irregular]	1.60 (0.97, 2.65)	0.07
Cystic tumour [yes]	0.18 (0.02, 1.30)	0.09
PVR^c^		
[<5%]	Reference	
[5%–25%]	1.21 (0.70, 2.10)	0.5
[≥25%]	1.89 (1.01, 3.56)	**0.048**

*Note*: Statistically significant findings are presented in bold.

Abbreviations: CI, confidence interval; HR, hazard ratio; PVR, parenchymal volume replacement.

^a^ Adverse pathology was defined as stage ≥pT3a, grade 3/4 or sarcomatoid/rhabdoid features.

^b^ Tumour‐related symptoms include flank/abdominal pain, gross haematuria, abdominal mass, weight loss >10% or loss of appetite, anaemia and severe fatigue.

^c^ Percent parenchymal volume replacement = [(parenchymal volume_contralateral_ − parenchymal volume_ipsilateral_) normalized by parenchymal volume_contralateral_] × 100%.

A nomogram to predict the probability of AP was constructed from the multivariable logistic regression model based primarily on patient/tumour characteristics and radiological features (Figure 2B). The ROC curve (AUC = 0.81, 95% CI: 0.78–0.84) indicated high discrimination of the model (Figure 2C, bias corrected C‐index = 0.80). A calibration curve with a bootstrap resampling (1000 repetitions) demonstrated high consistency between the predicted and actual probability of AP, supporting the accuracy of the predictive model (Figure 2D). Although the total R.E.N.A.L. score showed significant univariable associations with AP, the overall score was not included in the multivariable model because of collinearity with tumour size and its individual components. Each R.E.N.A.L. component was therefore evaluated individually (Figure S2). Among these, [N]earness was retained as an independent predictor in the final model. Conversely, [E]ndophyticity and [L]ocation components demonstrated only weak or inconsistent associations with AP and in some categories showed reverse directional relationships as shown in Figure S2; therefore, these components were excluded.

### 3.3 Predictive model for recurrence‐free survival

**FIGURE 3:**
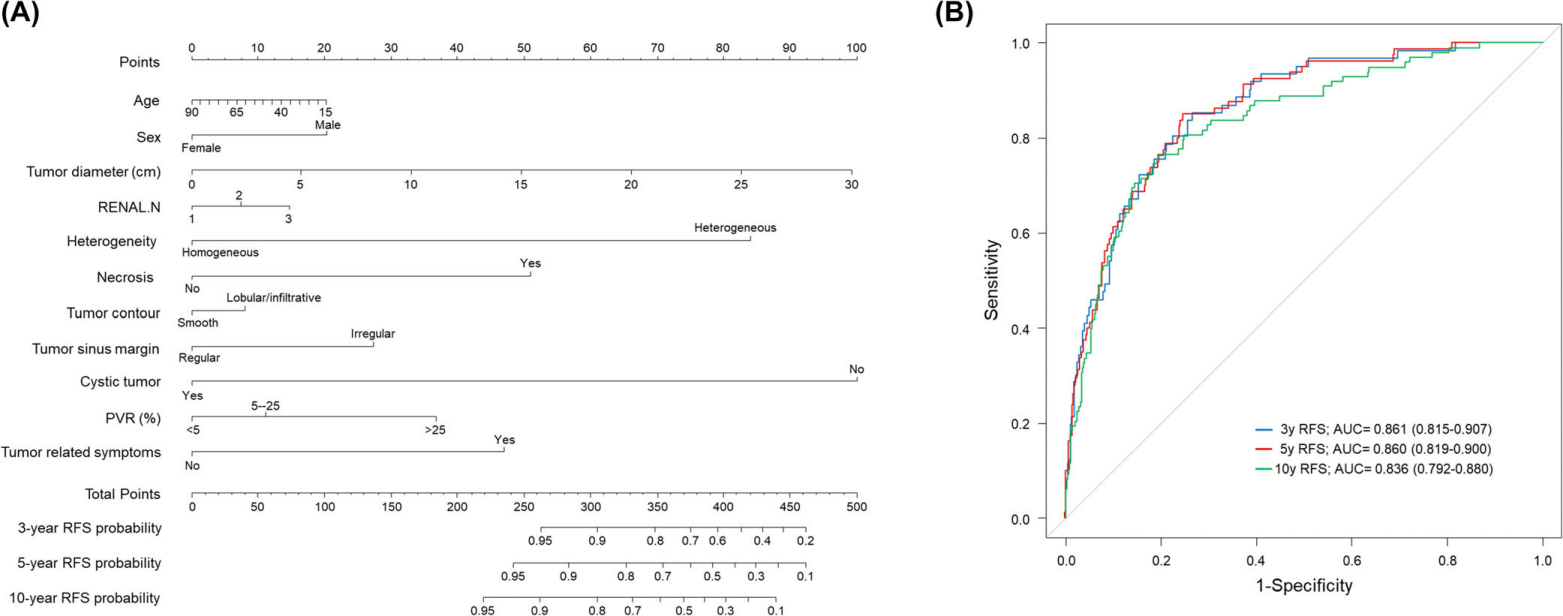
Nomograms to predict the probability of recurrence‐free survival. (A, B) Nomogram and ROC curves for predicting the probability of recurrence‐free survival at 3‐, 5‐ and 10‐year based on a Cox proportional hazards model. AUC = area under the curve, PVR = parenchymal volume replacement, R.E.N.A.L. = [R]adius, tumour size as maximal diameter; [E]xophytic/endophytic properties of tumour; [N]earness of tumour deepest portion to collecting system or sinus; [A]nterior [a]/posterior [p] descriptor; and [L]ocation relative to polar line, ROC = receiver operating characteristic.

The multivariable Cox regression model indicated that greater tumour size, heterogeneity, necrosis, prominent PVR (≥25%) and tumour‐related symptoms were significantly associated with reduced RFS (Table 3). Nomograms based on the predictive model for RFS resulted in apparent AUC values of 0.86 (3‐year RFS), 0.86 (5‐year RFS) and 0.84 (10‐year RFS), respectively (Figure 3). A bootstrap resampling (1000 repetitions) demonstrated high discrimination and calibration between the predicted and actual event rates, supporting the bootstrap internal validation of the predictive model (bias corrected C‐index = 0.81).

## 4 DISCUSSION

Preoperative assessment of the malignant potential of renal tumours is crucial for patient counselling to avoid overtreatment or unexpected upstaging. Previous studies have reported that 10%–30% of renal tumours managed with definitive surgery are benign.^25^, ^26^ In contrast, AP can be associated with higher postoperative recurrence rates and poor oncologic outcomes, particularly if not managed aggressively.^1^, ^2^, ^6^, ^27^ Therefore, appropriate preoperative oncologic risk stratification is required to improve patient counselling and outcomes. Numerous studies have aimed to predict AP using models based on patient characteristics and preoperative radiological features.^9^, ^10^, ^11^, ^12^, ^13^, ^14^, ^15^, ^16^, ^17^, ^18^ Our study provides a comprehensive evaluation of radiologic features to optimize preoperative oncologic risk stratification for patients with renal masses managed with definitive renal cancer surgery. We also include assessment of parenchymal volume replacement (PVR) in this algorithm,^20^, ^21^ which has not previously been used in such models.

Overall, AP, defined as stage ≥pT3a, tumour grade 3/4 or sarcomatoid/rhabdoid features, was found in 45% of patients and strongly associated with reduced RFS. Several radiographic features were evaluated and showed strong interrater agreement and significance on univariable analysis. On multivariable analysis, sex, R.E.N.A.L. [N]earness, heterogeneity, necrosis, sinus margin irregularity and increased PVR all independently associated with AP. Although many studies have indicated that tumour size is a strong predictive factor for AP,^9^, ^10^, ^11^, ^13^ interestingly, in our analysis, it was not a consistent independent predictor when multifaceted radiographic features were incorporated. Our nomogram, derived from a logistic regression model, showed a high C‐index (0.81) and strong calibration with bootstrap internal validation. Additionally, we developed nomograms to predict RFS using the Cox‐regression model and confirmed high predictive accuracies (C‐index 0.84–0.86). Given that few models have been reported to predict recurrence risk based primarily on preoperative radiographic features, our predictive model may be beneficial for counselling and in the decision‐making process regarding surgical procedures such as RN versus PN.

Renal mass biopsy (RMB) is currently considered a reference standard for presurgical assessment of renal mass histology. Societal guidelines strongly recommend RMB before TA, and in many patients planned for AS or PN, RMB can improve decision‐making regarding optimal tailored management.^1^, ^2^, ^5^, ^6^ RMB can reduce unnecessary surgical intervention for benign or indolent tumours, and if more aggressive tumour biology is suspected, it could influence decisions about RN versus PN or the potential role of wide margin PN versus tumour enucleation.^1^, ^2^, ^3^, ^7^ However, RMB is an invasive procedure with a 10%–15% non‐diagnostic rate, and it can be misleading due to tumour heterogeneity.^5^, ^6^ In some patients, RMB is not feasible due to unfavourable anatomic tumour location, cystic features raising concern about tumour spillage, patient intolerance of positioning for RMB or coagulopathy. Our predictive model can be considered in such patients, yet it may also be helpful for improved oncologic risk stratification in other patients where decision making is particularly challenging.

Previously, many models were developed to predict AP or prognosis of renal tumours.^9^, ^10^, ^11^, ^12^, ^13^, ^14^, ^15^, ^16^, ^17^ Some models predicting AP based on patient characteristics and tumour size have had limitations in accuracy, with C‐indices in the range of 0.65–0.7.^12^, ^13^ Several models for recurrence and cancer‐specific mortality reported high predictive accuracy (C‐index of 0.78–0.88).^17^, ^18^ However, these models are based on cohorts that exclude benign tumours and are not strictly based on preoperative factors alone, so their utility for preoperative counselling is compromised. Our model, developed from a large cohort that includes both benign and malignant cases, is highly adaptable and may serve as an alternative to RMB for preoperative oncologic risk stratification in select patients.

Recently, Wang et al. reported a nomogram that predicts AP by combining features of multi‐phase contrast‐enhanced CT and the neutrophil to lymphocyte ratio (NLR) to achieve high predictive accuracy (AUC = 0.85).^14^ In general, the cancer microenvironment produces inflammatory cytokines such as interleukins and interferon‐γ through upregulation of transcription factors in tumour cells.^28^ These inflammatory mediators recruit and activate various leukocytes including neutrophils and activated T cells. Inflammatory markers such as NLR, C‐reactive protein and albumin globulin ratio have been demonstrated to be strongly associated with malignant potential, including high tumour grade and sarcomatoid variant, and are strong predictors in either localized or metastatic disease.^29^ Nevertheless, the use of these markers can be complex because the relevant thresholds can vary widely depending on the diversity of the cohort on which the predictive models are constructed.

Artificial intelligence has also been explored in medical imaging to improve diagnostics, reduce subjectivity and inform decision‐making.^30^ Deep learning algorithms process raw images and compute output signals through multiple layers of transformation, including morphological characteristics, intensity‐related features and texture analysis. These technologies have shown promising results for predicting histology, tumour grade and differentiating benign lesions, such as fat‐poor angiomyolipoma and oncocytoma, from renal cancer, with accuracy rates approaching 90% in some studies.^30^ However, these algorithms are currently limited by concerns regarding generalizability and reproducibility, which need to be addressed. Future research should focus on further application of artificial intelligence to refine the predictive models developed in this study, transforming them into more powerful and clinically applicable tools.

In the current study, tumour size did not show significance as an independent predictor for AP after a comprehensive evaluation of a variety of other radiographic features. Based on analysis of 2770 cases, Frank et al. reported strong associations between increased renal tumour size and presence of malignancy versus benign pathology and increased tumour grade and stage.^9^ This study established tumour size as the strongest predictor of AP, which has been confirmed by other reports. Previous systematic review demonstrates increasing tumour size and male sex to be predictive of malignancy versus benign with robust effect sizes.^31^ More recently, Bhindi et al. demonstrated that increased tumour size is associated with aggressive pathology, as defined by histology and tumour grade, and other studies have shown an association between increased primary tumour size and a higher likelihood of metastasis.^10^ In our study, it is likely that there was confounding between tumour size and other radiographic features (e.g. R.E.N.A.L., PVR), with the latter 2 factors proving to be more impactful on multivariable analysis for prediction of AP.

Another unique feature of this study is that our model incorporates a relatively new entity, parenchymal volume replacement (PVR), which reflects the discordant loss of healthy parenchyma in the ipsilateral kidney due to invasive tumour growth.^20^, ^21^ As renal tumours grow, they can displace parenchyma or obliterate it in an aggressive manner. Two previous reports have demonstrated that PVR is associated with a decrease in preoperative ipsilateral renal function and is strongly correlated with AP.^20^, ^21^ Furthermore, prominent PVR, such as >25%, is associated with reduced RFS and cancer‐specific survival.^21^ Although PVR cannot be calculated for patients with atrophic or solitary kidneys because it uses the contralateral kidney as a control, it is a predictor that strongly reflects the malignant behaviour of renal tumours, and in our models, it proved to be an independent predictor of AP and reduced RFS. Our previous studies have demonstrated modest natural parenchymal volume discordance between the ipsilateral and contralateral kidneys which could potentially distort calculations of PVR; however, our statistical analyses accounted for this and confirmed the functional and oncologic relevance of PVR.^21^ Calculations of PVR are simple, facile and can be readily performed in the clinic setting (Figure 1).

Our study has limitations including a retrospective design within a single tertiary‐care centre, which could affect generalizability. Some patients were excluded due to lack of preoperative contrast‐enhanced imaging, solitary kidney or focal or global contralateral renal atrophy, which could introduce selection bias. While our study does not include an external validation cohort, it incorporates calibration curves with bootstrap resampling to internally validate the accuracy of the predictive models. Although inter‐observer agreement was generally strong, a small proportion of our cases required group review to reach consensus, and subjectivity remains a concern that will require further study and independent validation.

A strength of our study is that primary review of each case was performed by urologists suggesting that this approach can be implemented in the real‐world situation, with additional radiologic input, beyond the original read, only occasionally required. Other notable strengths of our study include the exclusive utilization of preoperative datapoints, which will improve patient counselling, and the comprehensive evaluation of multiple radiologic features and PVR, which is a relatively new and promising concept. Our predictive model also provides estimation for RFS with long‐term follow‐up.

## 5 CONCLUSIONS

Our study confirms that radiographic features are significantly associated with AP and reduced recurrence‐free survival after definitive renal cancer surgery. Interestingly, tumour size was not a consistent independent predictor when other imaging features were accounted for, likely due to confounding. Our predictive models provide precise preoperative risk stratification and may improve patient counselling and decision‐making. Furthermore, it may occasionally preclude the need for renal mass biopsy for some patients.

## AUTHOR CONTRIBUTIONS

*Research conception and design*: Akira Kazama, Carlos Munoz‐Lopez, Steven C. Campbell. Data acquisition: Akira Kazama, Carlos Munoz‐Lopez, Worapat Attawettayanon, Eran Maina, Nityam Rathi, Kieran Lewis, Anne Wong, Angelica Bartholomew, Erick M. Remer, Ryan Ward and Steven C. Campbell. *Statistical analysis*: Akira Kazama, Carlos Munoz‐Lopez, Amy S. Nowacki. *Data analysis and interpretation*: Akira Kazama, Carlos Munoz‐Lopez, Erick M. Remer, Ryan Ward, Steven C. Campbell. *Drafting of the manuscript*: Akira Kazama, Carlos Munoz‐Lopez, Steven C. Campbell. *Critical revision of the manuscript*: Rebecca A. Campbell, Jihad Kaouk, Samuel Haywood, Nima Almassi, Christopher J. Weight, Nick Heller, Shetal Shah, Erick M. Remer, Ryan Ward. *Approval of the final manuscript*: Akira Kazama, Carlos Munoz‐Lopez, Steven C. Campbell.

## CONFLICT OF INTEREST STATEMENT

None of the authors have any disclosures or conflict of interest to report.

## Supporting information


**Figure S1.** Representative CT images showing radiologic features of renal tumors that were evaluated in this study. Contrast CT images of radiologic features of renal tumors including enhancement (Hypovascular vs Hypervascular), heterogeneity (Homogeneous vs Heterogeneous), tumor contour (Smooth vs Lobular vs Infiltrative), tumor sinus margin (Regular vs Irregular), necrosis, and cystic tumor (cystic lesion >25%). The yellow markers indicated the tumor sinus margin.

**Figure S2.** Nomogram to predict the probability of adverse pathology based on a multivariable logistic regression model with inclusion of all R.E.N.A.L parameters. Subsequent models deleted the E., A., and L. components which did not substantially contribute to the predictive performance. Degree of enhancement was also excluded in the models. PVR=parenchymal volume replacement, R.E.N.A.L.=[R]adius, tumor size as maximal diameter; [E]xophytic/endophytic properties of tumor; [N]earness of tumor deepest portion to collecting system or sinus; [A]nterior [a]/posterior [p] descriptor; and [L]ocation relative to polar line.

**Table S1.** Interrater reliability for assessment of radiographic features.

**Table S2.** Individual data for each R.E.N.A.L. component.

**Table S3.** The R code used to generate logistic regression and Cox proportional hazards models, and time‐dependent ROC analyses.
