# Supplementary figures and images for: Preoperative predictors of adverse pathology and recurrence‐free survival for patients with renal masses
Source: BJUI Compass. 2026 Feb 27;7(3):e70175. doi: 10.1002/bco2.70175 (PMC12948496; doi:10.1002/bco2.70175)

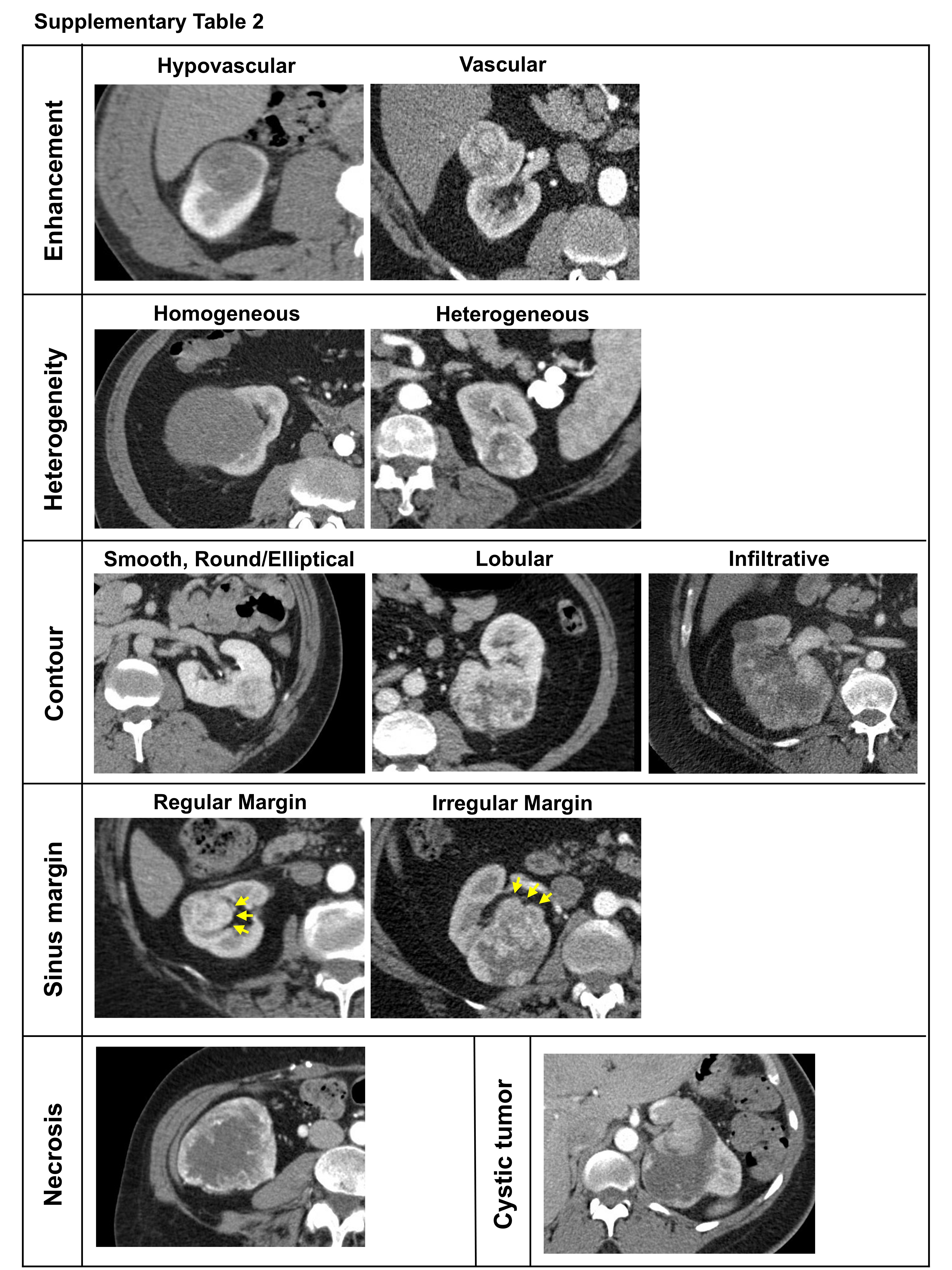

Supplement: Supplementary file 1 — Figure S1. Representative CT images showing radiologic features of renal tumors that were evaluated in this study. Contrast CT images of radiologic features of renal tumors including enhancement (Hypovascular vs Hypervascular), heterogeneity (Homogeneous vs Heterogeneous), tumor contour (Smooth vs Lobular vs Infiltrative), tumor sinus margin (Regular vs Irregular), necrosis, and cystic tumor (cystic lesion >25%). The yellow markers indicated the tumor sinus margin. [file BCO2-7-e70175-s001.jpg]

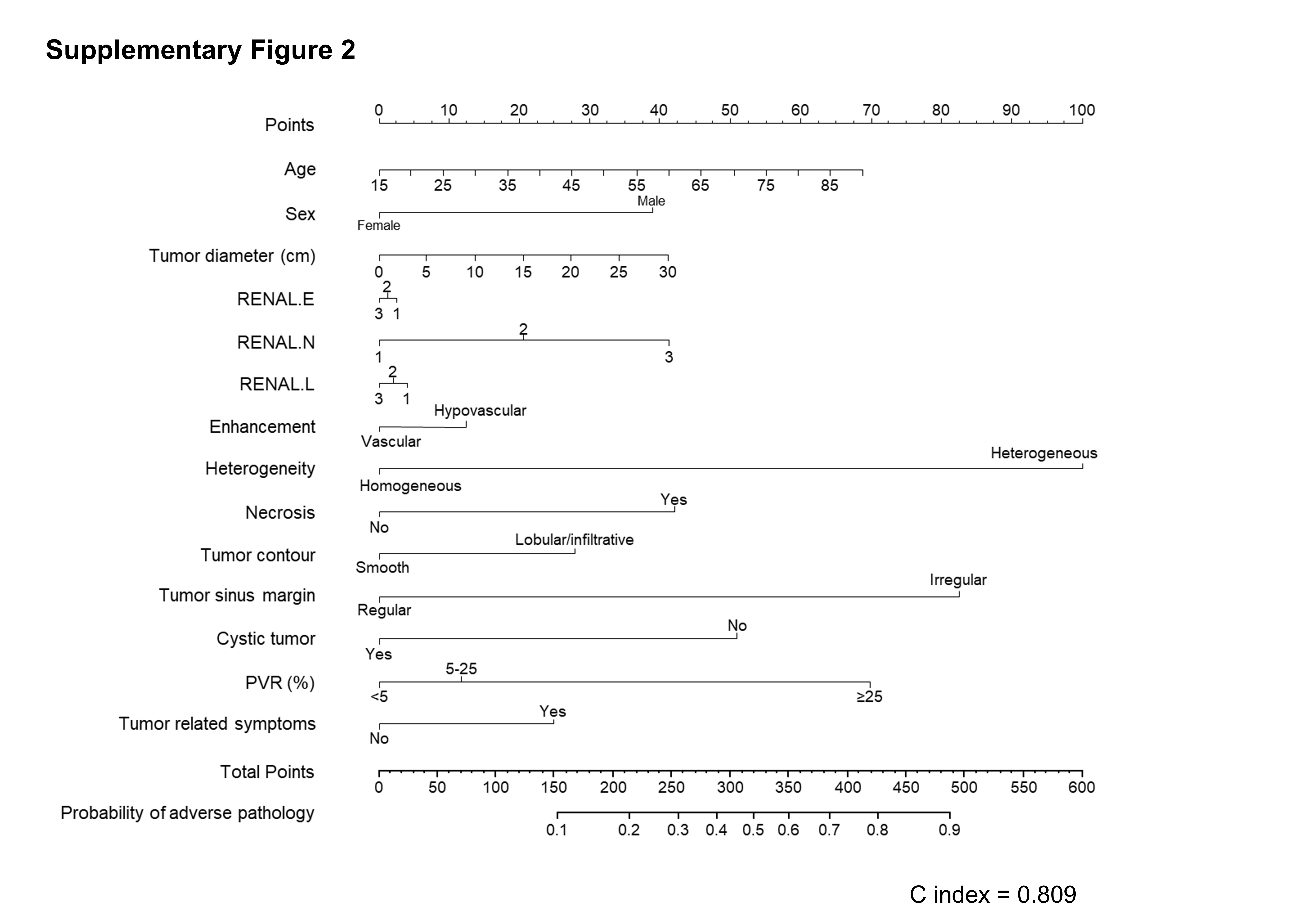

Supplement: Supplementary file 2 — Figure S2. Nomogram to predict the probability of adverse pathology based on a multivariable logistic regression model with inclusion of all R.E.N.A.L parameters. Subsequent models deleted the E., A., and L. components which did not substantially contribute to the predictive performance. Degree of enhancement was also excluded in the models. PVR=parenchymal volume replacement, R.E.N.A.L.=[R]adius, tumor size as maximal diameter; [E]xophytic/endophytic properties of tumor; [N]earness of tumor deepest portion to collecting system or sinus; [A]nterior [a]/posterior [p] descriptor; and [L]ocation relative to polar line. [file BCO2-7-e70175-s003.jpg]
