# Supplementary material for: Preoperative predictors of adverse pathology and recurrence‐free survival for patients with renal masses
Source: BJUI Compass. 2026 Feb 27;7(3):e70175. doi: 10.1002/bco2.70175 (PMC12948496; doi:10.1002/bco2.70175)
Supplement: Supplementary file 3 — Table S1. Interrater reliability for assessment of radiographic features. [file BCO2-7-e70175-s004.docx]

**Supplementary Table 1. Interrater reliability for assessment of radiographic features**

| Radiological parameter | Kappa value | 95% CI |
| --- | --- | --- |
| Enhancement | 0.88 | 0.83, 0.94 |
| Heterogeneity | 0.95 | 0.91, 0.99 |
| Contour | 0.94 | 0.90, 0.98 |
| Sinus margin | 0.92 | 0.87, 0.97 |
| Necroiss | 0.93 | 0.87, 0.99 |
| Cystic tumor | 0.88 | 0.80, 0.97 |

Abbreviation; CI=confidence interval,
