# Supplementary material for: Preoperative predictors of adverse pathology and recurrence‐free survival for patients with renal masses
Source: BJUI Compass. 2026 Feb 27;7(3):e70175. doi: 10.1002/bco2.70175 (PMC12948496; doi:10.1002/bco2.70175)
Supplement: Supplementary file 4 — Table S2. Individual data for each R.E.N.A.L. component. [file BCO2-7-e70175-s002.docx]

**Supplementary Table 2. Individual data for each R.E.N.A.L. component**

| Parameter | Non-adverse pathology^a^  (N=409) | Adverse pathology(AP)^a^  (n=339) | p value |
| --- | --- | --- | --- |
| RENAL. [R]adius (%) |  |  |  |
| 1 | 250 (61.1) | 98 (28.9) | <0.01 |
| 2 | 123 (30.1) | 119 (35.1) |  |
| 3 | 36 ( 8.8) | 122 (36.0) |  |
| RENAL. [E]ndophyticity (%) |  |  |  |
| 1 | 136 (33.3) | 76 (22.4) | <0.01 |
| 2 | 196 (47.9) | 184 (54.3) |  |
| 3 | 77 (18.8) | 79 (23.3) |  |
| RENAL. [N]earness (%) |  |  |  |
| 1 | 164 (40.1) | 50 (14.7) | <0.01 |
| 2 | 74 (18.1) | 31 ( 9.1) |  |
| 3 | 171 (41.8) | 258 (76.1) |  |
| RENAL. [L]ocation (%) |  |  |  |
| 1 | 151 (36.9) | 67 (19.8) | <0.01 |
| 2 | 144 (35.2) | 109 (32.2) |  |
| 3 | 114 (27.9) | 163 (48.1) |  |

Summary statistics are reported as either number (%).

Abbreviations; R.E.N.A.L.= [R]adius, tumor size as maximal diameter; [E]xophytic/endophytic properties of tumor; [N]earness of tumor deepest portion to collecting system or sinus; [A]nterior [a]/posterior [p] descriptor; and [L]ocation relative to polar line.

^a^ Adverse pathology was defined as stage ≥pT3a, grade 3/4, or sarcomatoid/rhabdoid features.
