# Supplementary material for: Preoperative predictors of adverse pathology and recurrence‐free survival for patients with renal masses
Source: BJUI Compass. 2026 Feb 27;7(3):e70175. doi: 10.1002/bco2.70175 (PMC12948496; doi:10.1002/bco2.70175)
Supplement: Supplementary file 5 — Table S3. The R code used to generate logistic regression and Cox proportional hazards models, and time‐dependent ROC analyses. [file BCO2-7-e70175-s005.docx]

**Supplementary Table 3. The R code used to generate logistic regression and Cox proportional hazards models, and time-dependent ROC analyses.**

**Logistic regression model for predicting Adverse Pathology**

library(rms)

f <- lrm(Adverse.pathology ~ Age + Sex + Tumor.diameter + RENAL.N + Heterogeniry + Necrosis + Tumor.contour + Tumor.sinus.margin Cystic.tumor + PVR + Tumor.related.symptoms , 　data =Dataset, x=TRUE, y=TRUE)

f

ddist <- datadist(Dataset)

options(datadist='ddist')

nom <- nomogram(f, fun=plogis, funlabel="Probability", lp=F)

plot(nom, cex=0.9, cex.axis = 0.8,

lmgp = 0.2, )

**Cox regression hazards model for Recurrence-free survivals**

library(rms)

library(survival)

f<- cph(Surv(RFS , Recurrence ==1)~ Age + Sex + Tumor.diameter + RENAL.N + Heterogeniry + Necrosis + Tumor.contour + Tumor.sinus.margin Cystic.tumor + PVR + Tumor.related.symptoms , 　data =Dataset, x=TRUE,y=TRUE,surv=TRUE)

f

dd <- datadist(Dataset)

options(datadist = "dd")

surv <- Survival(f)

nom <- nomogram(f, fun=list(function(x) surv(36, x), function(x) surv(60, x),

function(x) surv(120, x)), lp=F, funlabel=c("3-year RFS", "5-year RFS", "10-year RFS"),

maxscale=100, fun.at=c(0.95, 0.9, 0.8, 0.7, 0.6, 0.5, 0.4, 0.3, 0.2, 0.1))

plot(nom, cex=1, cex.axis = 1, lmgp = 0.2,)

**Time-dependent ROC for Recurrence-free survivals**

library(timeROC)

tr <- timeROC(T = Dataset$RFS, delta = Dataset$Recurrence, marker = lp, cause = 1, times = c(36, 60, 120),

iid = TRUE )

tr

plot(tr, time = 36, col = 1, title = TRUE)

plot(tr, time = 60, col = 2, add = TRUE)

plot(tr, time = 120, col = 3, add = TRUE)

PVR; parenchymal volume replacement, ROC; receiver operating characteristic
